# The Role of Growth Hormone in Mesenchymal Stem Cell Commitment

**DOI:** 10.3390/ijms20215264

**Published:** 2019-10-23

**Authors:** Simona Bolamperti, Francesca Guidobono, Alessandro Rubinacci, Isabella Villa

**Affiliations:** 1 Bone Metabolism Unit, Division of Genetics & Cell Biology, IRCCS Ospedale San Raffaele, 20132 Milan, Italy; bolamperti.simona@hsr.it (S.B.); rubinacci.alessandro@hsr.it (A.R.); 2 Department of Medical Biotechnology and Translational Medicine, University of Milan, 20129 Milan, Italy; francesca.guidobono@unimi.it

**Keywords:** growth hormone, cell differentiation, bone repair, regenerative medicine

## Abstract

Growth hormone (GH) is best known for its prominent role in promoting prepubertal growth and in regulating body composition and metabolism during adulthood. In recent years, the possible role of GH in the modulation of mesenchymal stem cell (MSC) commitment has gained interest. MSCs, characterized by active self-renewal and differentiation potential, express GH receptors. In MSCs derived from different adult tissues, GH induces an inhibition of adipogenic differentiation and favors MSC differentiation towards osteogenesis. This activity of GH indicates that regulation of body composition by GH has already started in the tissue progenitor cells. These findings have fostered research on possible uses of MSCs treated with GH in those pathologies, where a lack of or delays in bone repair occur. After an overview of GH activities, this review will focus on the research that has characterized GH’s effects on MSCs and on preliminary studies on the possible application of GH in bone regenerative medicine.

## 1. Introduction

Mesenchymal stem cells (MSCs), first detected by Friedenstein and colleagues [1] in the stromal compartment of bone marrow, are widely believed to constitute a reserve to replace damaged and aged cells. MSCs have the capacity to differentiate into a variety of cell types, including adipocytes, osteoblasts, chondrocytes, and myoblasts. Due to their multipotency, recent research has highlighted their potential usefulness in tissue-regenerative cell therapies. Considering their multiple differentiation potential and their ability to secrete a broad variety of biological active factors, the identification of factors that can regulate commitment is essential for the therapeutic use of MSCs. Among all these factors, the modulatory activity of growth hormone (GH) on MSC differentiation has gained interest in recent years. This review will focus on GH activity on MSC differentiation and on the possible application in regenerative medicine.

## 2. Growth Hormone Synthesis and Secretion

GH is a single-chain peptide of 191 amino acids produced by the anterior pituitary, and its synthesis and secretion is tightly regulated by the hypothalamus through the release of growth hormone-releasing hormone (GHRH), which stimulates GH secretion through gene transcription and through the release of somatostatin, which inhibits GH secretion from the pituitary. Thus, regulation of GH levels by the hypothalamus results from the balance between these two factors. The secretion of GH is pulsatile, and displays a circadian rhythm. It shows an episodic nature during the day with elevated nocturnal peaks during sleep. GH stimulates the synthesis and the secretion of insulin-like growth factor I (IGF-I) by the liver which, in turn, inhibits GH secretion directly in the pituitary and indirectly by stimulating the release of somatostatin [2]. Recently, ghrelin was discovered, which is a peptide produced from the gastrointestinal tract that synchronizes with GHRH for stimulating GH secretion [3,4], and is the endogenous ligand for the GH secretagogue receptor, a G-protein-coupled receptor [5]. Several other factors and hormones regulate GH secretion: sex steroids and thyroid hormones stimulate GH secretion whereas corticosteroids inhibit it. Changes in body composition and reduced physical activity suppress GH secretion [6,7]. GH may also act locally as an autocrine/paracrine factor as GH gene expression is not confined to the pituitary gland. In fact, GH is present in several other tissues, such as neural, reproductive, immune, ocular, cardiovascular, muscular, dermal, and skeletal tissues. These local productions do not influence circulating levels of GH [8,9].

Besides the rate of pituitary secretion and glomerular clearance, the bioavailability of circulating GH depends on its binding to GH-binding protein (GHBP) [10,11]. Normally, about 45% of circulating GH is bound to GHBP. GHBP is generated by proteolytic cleavage of the extracellular domain of the GH receptor (GHR) or by mRNA splicing [12]. The main source of GHBP is the liver, which produces at least 75% of GHBP [13]. Nevertheless, synthesis by other tissues, such as muscle and adipose tissue, may contribute to the circulating levels of GHBP [14,15]. Serum levels of the GHBP serve as a marker of GHR expression and the GH responsiveness of tissues [12]. The function of the GH-binding protein is incompletely understood; it could modulate the activity of GH either by prolonging its half-life or by reducing its bioavailability.

The level of circulating GH undergoes changes during different stages of life: during early life to promote growth development and during puberty and adult life to exert metabolic activities [16]. During aging, there is a gradual decline in GH levels, however, little is known on the cause–effect relationship between the decline of GH and senescent changes in body composition. In normal adults, the senescent decline in GH levels is paralleled by a decline in serum IGF-I, suggesting a downregulation of the GH–IGF-I axis [17]. GH–IGF-I axis dysregulation could contribute to the age-related progressive loss of muscle mass and osteopenia [18]. Moreover, the secretion of GHRH by the hypothalamus is decreased and the secretion of somatostatin is increased. Studies to elucidate these aspects will be of interest for the implication for potential intervention as the decline in GH and IGF-I is accompanied by progressive loss of muscle mass and by changes in body composition.

## 3. Growth Hormone Intracellular Signaling

To exert its action, GH binds to a specific receptor which belongs to the class I cytokine receptor family [19]. It is composed of three main domains: an extracellular domain, a transmembrane domain, and an intracellular domain. The extracellular domain has two structural regions which contain the ligand binding site and receptor dimerization site. A single transmembrane domain goes across the cell membrane and is involved in the stabilization of the dimer. The intracellular domain contains two well conserved regions that are involved in signal transduction and receptor internalization. Functional GHRs are present in the cell plasma membrane as dimers that are stabilized through interactions in the transmembrane domain [20]. One GH molecule binds to the extracellular domain of the GHR dimer [21,22] and induces a conformational change in the extracellular domain and rotation of the transmembrane domain that are essential for the activation of the intracellular signaling cascade. [20].

The classical intracellular signaling induced by GH is the phosphorylation of Janus kinase 2 (JAK2), with subsequent phosphorylation of the signal transducers and activators of transcription (STATs). STAT proteins then translocate into the nucleus, where they bind to specific DNA motifs within the promoter regions to initiate transcription of GH-responsive genes. GH activation of the JAK/STAT pathway is negatively regulated by phosphotyrosine phosphatases (PTPs) [23] and the suppressors of cytokine signaling (SOCS) [24,25]. After GH binding and intracellular signaling activation, GHR is internalized and degraded via an ubiquitin-dependent mechanism [26,27].

**Figure 1:**
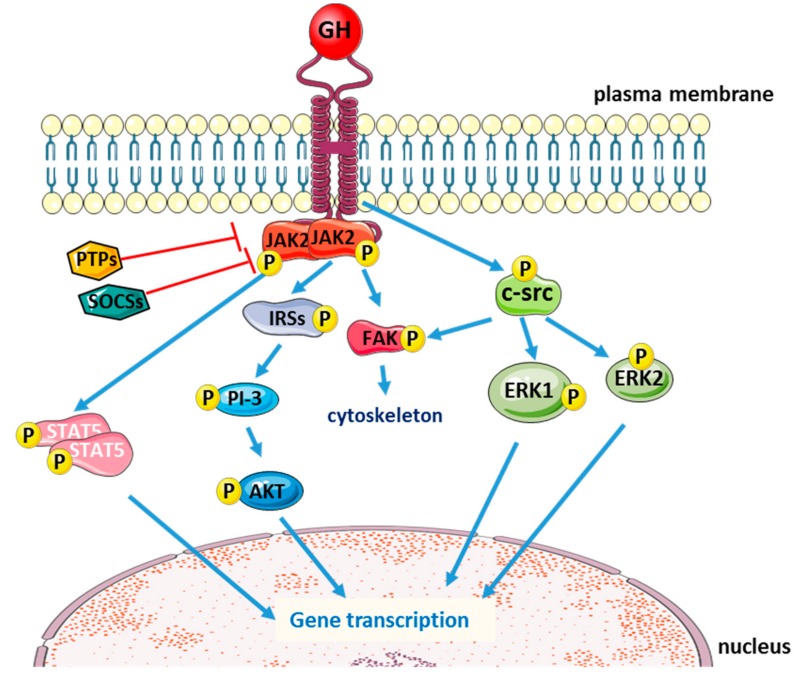
Schematic representation of the main intracellular signaling pathways activated by the binding of growth hormone (GH) to its receptor (blue arrows: activation; red T-arrows: inhibition).

GH binding could also lead to JAK2-dependent phosphorylation of insulin receptor substrate (IRS) proteins 1, 2, and 3, thus activating the PI-3 kinase/Akt pathway [28,29,30,31] and to focal adhesion kinase (FAK) activation that promotes GH-induced reorganization of the cytoskeleton in a variety of cell types [32,33]. FAK activation seems to be induced not only by JAK2 [34], but also independently from JAK2 via the c-Src family of protein kinases [35]. In fact, besides JAK2/STAT5a,b pathway, which is crucial for GH-induced changes in metabolic function and body growth [36,37,38], GH binding to GHR could also activate the src family kinases with subsequent phosphorylation of extracellular signal-regulated kinase (ERK) 1,2 (Figure 1). The ratio between activation of JAK2 and src family kinases depends on the cell type [39].

Intriguingly, nuclear localization of GHR has been observed in cancer cells [40,41]. Nuclear localization of a transmembrane receptor has been demonstrated also for other receptors, e.g., epidermal growth factor and fibroblast growth factors, and often induces the transformation of the cell. It is likely that nuclear localization of GHR sensitizes cells to autocrine GH action, which induces altered expression of the transformation and proliferation genes [42].

## 4. Growth Hormone Activities

GH exerts important biological and physiological effects, both by acting directly on the target tissues and indirectly through the secretion of IGF-I by the liver. Before puberty, GH promotes skeletal and lean tissue growth and differentiation. Later in life, when growth is no longer a physiological urgency, GH is involved in the regulation of body composition; in adults, GH has significant effects on the maintenance of bone and skeletal muscle mass, on adiposity, and on metabolism.

The anabolic effects of GH on skeletal development are the best known. GH is responsible for increasing height during childhood and is important for the maintenance of bone mass and homeostasis in adults [43]. Besides IGF-I-mediated effects, GH directly stimulates osteoblast proliferation and activity [44], promoting bone formation. It also stimulates osteoclast differentiation and activity, increasing bone resorption. The balance of these two actions results in an increase in the overall rate of bone remodeling, with a net effect of bone accumulation [45,46]. Most of the information on GH activity has been obtained by studying alterations (deficiency/excessive secretion) in GH levels, both in human pathologies and in mice models. Patients with congenital GH deficiency (GHD) have severe postnatal growth retardation in early infancy (during or after the first year of life) and a markedly short height in adulthood. Their phenotype also includes trunk obesity, acromicria, delayed dentition, and delayed skeletal maturation causing osteopenia [47,48]. In adult-onset GHD, the absence of GH causes low bone turnover osteoporosis, leading to increased fracture risk and a rise in the adiposity of the bone marrow cavity of long bones. GH replacement therapy reverts this severe skeletal phenotype. In humans, recombinant human GH (rhGH) supplementation has been approved by the Food and Drug Administration as a pharmacological treatment for GHD subjects since 1985 [49].

Skeletal muscle is a target tissue for GH, where it induces muscle hypertrophy by increasing protein synthesis and reducing protein oxidation [50]. This effect is time-dependent, as after chronic treatment with GH in GHD subjects, the body protein metabolism reaches a new balance when lean body mass reaches a plateau [51]. Muscle function is dependent on the availability of metabolism fuel to synthetize ATP [52]. By favoring lipid catabolism and gluconeogenesis, GH increases free fatty acid and glucose levels. Free fatty acids are the major source of energy rather than glucose, especially during fasting. GH, by providing substrates for the production of energy, induces an anabolic effect on muscle mass. Furthermore, GH, independently from IGF-I, increases the size of differentiated myotubes in a cell-autonomous manner, and favors myoblast fusion when nascent myotubes are present and develop into mature myotubes [53]. Since GH uses JAK2/STATs signaling to induce many of its direct actions as well as the stimulation of IGF-I production, several contrasting results have been published as it is difficult to attribute specific independent roles to GH or IGF-I [54].

Circulating levels of GH are negatively associated with adipose tissue mass in both humans and mice. In subjects affected by acromegaly, all fat depots are reduced, with the greatest extent in the visceral depot [55]. Accordingly, in a transgenic mouse model constitutively expressing bovine GH (bGH mice), fat mass is decreased in all depots, with a more pronounced reduction in the subcutaneous depot [56]. Interestingly, bGH mice fed with high fat diets are also resistant to diet-induced obesity, exhibiting preferential accumulation of lean tissue instead of adipose tissue [57]. In humans, GHD is associated with increased lipid deposits. Interestingly, when GHD subjects are under GH therapy, the extra fat mass, in particular, the intra-abdominal fat, reverts to ranges of nondeficient subjects [58]. GH seems also to reduce both abdominal fat [59] and total fat mass in obese subjects characterized by lower GH secretion, with fewer GH secretory pulses and shorter half-life duration [60]. Generally, the levels of GH are negatively associated with the levels of leptin and adiponectin as observed in acromegalic subjects and bGH mice [61,62].

GH has a crucial role in carbohydrate metabolism; it favors gluconeogenesis, hyperinsulinemia, and insulin resistance. The mechanism underlying insulin resistance is complex: it could be due to GH interfering with insulin intracellular signaling as well as to the GH-induced increase of free fatty acids. However, the diabetogenic action of GH is evident under conditions of GH excess, either in acromegalic subjects or during hormone replacement therapy.

Regarding body protein metabolism, GH stimulates protein synthesis and reduces protein catabolism. In the normal fed state, GH promotes anabolism by increasing the production of insulin and IGF-I, thus increasing muscle mass at the expense of adipose tissue [63]. During periods of fasting, GH secretion increases and promotes the use of fatty acids instead of glucose as a source of energy, thus protecting against excessive catabolism of proteins and, at the same time, preserves glucose for the brain [63,64]. Thus, when nutrient intake is reduced, GH shifts cell consumption from carbohydrates and protein catabolism to the use of lipids, thereby preserving vital protein stores.

## 5. Mesenchymal Stem Cells: A Brief Overview

The cells known as “mesenchymal stem cells” or “multipotent mesenchymal stromal cells” (MSCs) are widely believed to constitute a reserve to replace damaged and aged cells, and to be potentially useful in tissue-regenerative cell therapies [65,66,67,68]. In the living organism, these progenitor cells are responsible for the cell replacement required during normal turnover for the maintenance of tissue integrity. As MSCs can be obtained in suitable amounts from different types of tissue in adults, they are the optimal candidates for applications in repairing/regenerating damaged tissue. Furthermore, MSCs—having reduced immunogenic potential and immunosuppressive activity—are also suitable for allogenic stem cell therapy [69,70].

In order to identify MSC populations, the International Society for Cellular Therapy (ISCT) has defined three criteria that should be concurrently present: cells should adhere to plastic; they should be able to differentiate into chondrocytes, osteoblasts, and adipocytes under standard in vitro differentiation conditions; and they should be positive for the surface markers CD105, CD73, and CD90, and negative for CD45, CD34, CD14, CD11b, CD79α, CD19, and HLA-DR [71], in order to exclude hematopoietic cells which may contaminate MSC cultures. In bone marrow MSCs (BM-MSCs), several other combinations of markers have been detected and proposed, so far [72,73], at different stages of differentiation. Up to now, a broad consensus on the combination of markers that define human BM-MSC has not yet been achieved.

For a long time, bone marrow has been the main source of MSCs, although the percentage of MSCs compared to hematopoietic precursors is low. It should be noted that in bone marrow, MSCs represent less than 0.01% of the overall mononucleated cells [74]. Furthermore, the methods for harvesting MSCs from bone marrow (bone biopsy at the iliac crest or at the breastbone) are painful, limiting the use of BM-MSCs in research and clinical settings. After these initial findings in adult bone marrow, MSCs were discovered in almost all adult tissues [75,76,77]. Among all tissues, adipose tissue and umbilical cord turned out to be the most promising source for adult MSCs. Adipose tissue was found to be an especially rich source of MSC, with up to 3% stem and progenitor cells in its stromal vascular fraction. The specific location of the MSC population within intact adipose tissue has been proposed to be in a perivascular location proximate to pericytes and endothelial cells [78]. It has also been hypothesized that blood vessels in virtually all organs and tissues possess MSCs in their perivascular niche [79]. Since adipose tissue MSCs (AT-MSCs) can be obtained by minor invasive procedure and in large amounts, they have become important candidates in autologous and allogenic stem cell-based therapies. Besides adipose tissue, the umbilical cord has proven to be a suitable source for MSCs that are characterized by a higher degree of multipotency than BM-MSC or AT-MSC [80].

Besides the capacity of MSCs to differentiate into distinct mature cell types, MSCs display trophic activity. In fact, MSCs that are recruited to sites of injury or disease secrete bioactive factors [75] that are immunomodulatory and trophic [81], and that are crucial for organization around the microenvironments of tissue injury. The study by Lin et al. [82] showed that infused auto or allogenic MSCs appear to be recruited to sites of injury or inflammation [82]. In this case, MSCs rarely differentiate into the damaged tissue [75], but they secrete bioactive factors [83] to stimulate site-specific and tissue-specific resident stem cells to build new tissue [84,85].

In vitro MSCs have a limited lifespan as any normal somatic cell. Senescent MSCs display a typical morphology, characterized by enlarged and irregular cell shapes and, ultimately, they stop proliferating [86]. Many studies in both humans and rodents have reported a decline in the frequency of CFU-Fs (mesenchymal colony-forming units) related to the biological age of the bone marrow [87]. By evaluating MSCs derived from bone marrow of human subjects in an age range from 17 to 90 years, Zhou et al. [88] found that MSCs from older subjects were more apoptotic with higher expression of p53 and its pathway genes p21 and BAX, and also produced fewer osteoblasts than the younger subjects [88]. Thus, donor age and the analysis of in vitro senescence in MSCs is crucial for basic research as well as for quality control in cellular therapy. Considering that during aging, the reduced number of MSCs could play a role in the maintenance of a proper tissue turnover and in the repair/regeneration of damaged tissue, the possibility to deliver MSCs in situ could improve the process of repair and the quality of life of the senile population.

## 6. Growth Hormone and Mesenchymal Stem Cells

**Figure 2:**
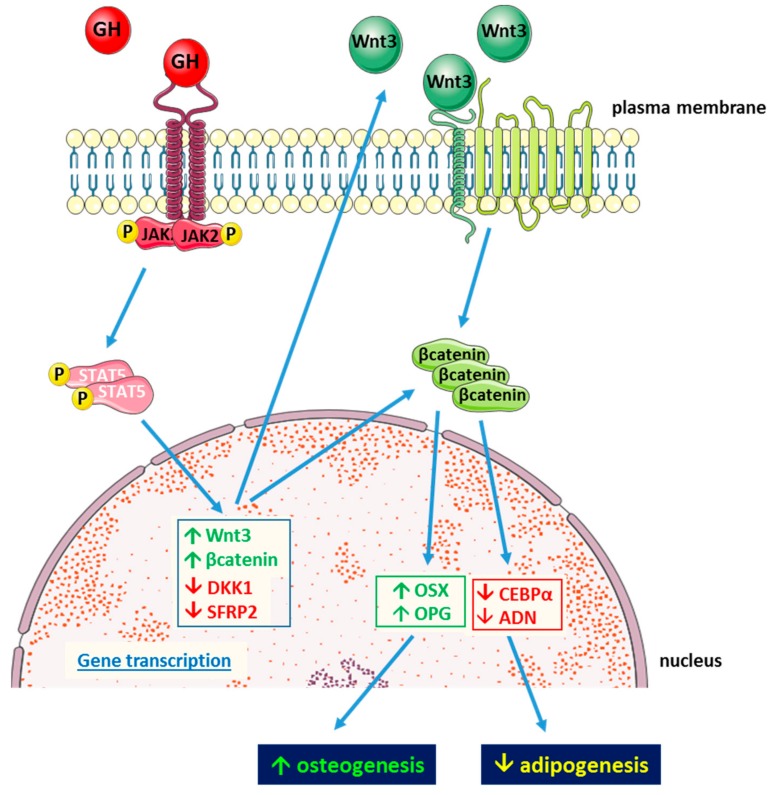
Schematic representation of antiadipogenic action of GH in human mesenchymal stem cells (MSCs) derived from trabecular bone (blue arrows indicate related effects).

To better understand the mechanisms underlying the effects of GH on its target tissues, studies on GH activity in MSC have focused on BM- and AT-MSCs derived from humans and mice. Studies on AT-MSC isolated from a mouse knockout for GH receptor (GHRKO) revealed that these cells display increased differentiation towards adipocytes with increased levels of adipogenesis-related genes (peroxisome proliferator-activated receptor γ (*PPARG*), fatty acid binding protein 4 (*FABP4*), adiponectin (*ADIPOQ*)) and intracellular lipid droplet accumulation compared to wild type controls. On the other hand, AT-MSCs isolated from mice transgenic for bovine GH (bGH) display less lipid accumulation and lower levels of adipogenesis-related genes than both wild type and GHRKO mice. As in these cells, transcription of *AXIN2* gene, which is a target of Wnt/β-catenin signaling, is increased, and the authors suggest a possible role of this pathway in the modulation of MSC fate by GH [89]. Although the hypothesis of Wnt signaling involvement relies on the evaluation of mRNA expression of only one gene, it is generally acknowledged that Wnt signaling plays a key role in the commitment of MSC by favoring osteogenesis at expense of adipogenesis [90,91]. Inhibition of MSC differentiation towards adipocytes by GH has been recently confirmed in MSCs derived from human trabecular bone. Human BM-MSC express GHR and respond to GH via JAK2/STAT5 intracellular signaling. In these cells, physiological levels of GH inhibit cell lipid accumulation after 14 days of culture in lipogenic medium. The expression of the adipogenic genes, C/EBPα and adiponectin, and the lipogenesis-related enzymes lipoprotein lipase and acetylCoA carboxylase are reduced, whereas the osteogenic factors Osterix and osteoprotegerin are increased by GH. In parallel, Wnt inhibitors are reduced, Wnt activator increased, and β-catenin accumulates in the nucleus. To mechanistically demonstrate the involvement of the Wnt pathway in the antiadipogenic action of GH, β-catenin was silenced. The blockage of Wnt intracellular cascade was shown to prevent GH from inhibiting the adipogenic differentiation of these precursors (Figure 2). This study further supports the hypothesis that GH antiadipogenic effect involves Wnt signaling activation [92].

The decline of GH activity on MSC could be involved in the increase of the fat component of bone marrow that occurs during aging. In fact, it could be hypothesized that when GH levels decline as in aging, MSC differentiation is shifted towards adipogenesis at the expense of osteoblastogenesis and bone formation, thus contributing to the reduction of bone mass observed in the elderly [92].

Recently, Jia and colleagues [93] showed that bovine rGH has a role in the commitment of the C3H10T1/2 cell line. These cells are multipotent cells derived from an early mouse embryo, and are considered a suitable cell model for MSCs. In this study, the authors showed that GH increases the number of myotubes formed in presence of 5′-azacytidine, which is a chemical analogue of the nucleoside cytidine that has been previously demonstrated to promote myogenesis [94]. Furthermore, GH treatment reduces by 50% the number of adipocytes formed in C3H10T1/2 cells treated with 5′-azacytidine compared to C3H10T1/2 cells treated with 5′-azacytidine alone [93].

GH can synergize with other growth factors to modulate MSC commitment. Huang et al. (2012) showed that combined treatment of GH and BMP9 induces a greater expression of early and late markers of osteogenesis in a murine MSC cell line. Furthermore BMP9, which is a strong activator of osteogenesis of murine multipotent progenitors, directly stimulates GH gene transcription suggesting a possible autocrine activity of GH in these cells. Interestingly, in an ectopic bone formation model, BMP9 and GH co-stimulation of a murine MSC cell line induces greater ectopic mature bone formation than BMP9 alone, and this effect is inhibited by silencing GH expression or by using inhibitors of the JAK/STAT signaling pathway [95].

In summary, it appears that the main role of GH in MSC modulation is to inhibit MSC differentiation towards adipocytes via activation of the Wnt signaling pathway, which is a major player in osteogenic commitment of MSCs. Furthermore, GH promotes osteogenic differentiation of MSCs, and this effect is enhanced in combination with other growth factors. Overall, these findings indicate that MSC, independently from tissue origin, are a target for GH activity that, by modulating their commitment, could influence body composition.

## 7. Growth Hormone and Mesenchymal Stem Cells in Bone Regenerative Medicine

The original rationale for the application of MSCs in tissue regeneration is the restoration of damaged cells and their differentiation into tissue specific cells for de novo tissue formation. Recently, the idea of activating GH signaling to potentiate MSC osteogenic differentiation has started to be explored. In bone prosthetic surgery, MSCs respond to metallic surfaces by producing soft tissue instead of bone, which can impair the expected outcome [96]. Therefore, the possibility to have osteogenic factors to be delivered in situ could be of great advantage in this clinical issue. Previous studies in a model of bone repair in rabbits demonstrated that locally administered GH has positive effect on bone formation without having the undesirable effect of GH systemic administration. GH locally released via calcium phosphate biomaterial implanted in rabbit femurs induces quicker repair of the bone damage [97]. A positive effect of GH was also observed in the newly formed peri-implant bone in rabbit tibiae [98]. To better characterize the mechanisms underlying this beneficial effect of GH in bone repair and considering that MSCs play a central role in tissue repair, recent research has focused on GH activity on these cells. It has been demonstrated that custom-engineered materials (polymethylmethacrylate, polycaprolactone) with randomly placed nanopits with a diameter and depth of 50 nm (NSQ50) favors osteogenic differentiation of human MSCs in the absence of osteogenic differentiating medium [96]. Wang et al. [99] used this type of growth surface to investigate the osteogenic effects of GH on human BM-MSC differentiation. During the spontaneous differentiation of MSC on these surfaces, the authors detected the expression of GHR, and found that administration of recombinant human GH (rhGH) leads to an enhancement of osteogenesis as determined by the increased expression of the late osteogenic marker osteopontin and von Kossa staining for mineralization [99].

Further evidence has been provided by da Silveira Gerzson and colleagues [100], who produced a scaffold of poly(lactic-co-glycolic acid) (PLGA) containing rhGH that permitted slow release of the hormone. Murine BM-MSCs plated on the scaffold with the GH-polymer combination had higher cell proliferation compared to the cells plated on the sole scaffold. The authors also noted that GH increased the roughness of the polymer, thus favoring cell adhesion. The use of PLGA scaffolds with the addition of rhGH could be practicable in conditions of maxillofacial bone defects, possibly also in combination with bone substitutes (hydroxyapatite, calcium phosphate, and beta-tricalcium phosphate). Considering GH osteogenic activity on MSCs in vitro, it could not be excluded that local release of GH could also activate resident MSC.

Despite these promising pilot studies, there are still several limitations in the application of these techniques in humans. So far, there are discrepancies in the results obtained from the in vivo studies, where it seems unlikely that exogenously supplied MSC could differentiate into osteoblasts but, more likely, they could activate the resident MSCs to repair bone damage by secreting growth and immunomodulatory factors. Moreover, the microenvironment of the recipient could play an important role. In particular, inflammation state, age, gender, etc. could influence the outcome of the therapeutical approach. Nevertheless, the results obtained with GH, although sparse, seems promising for the resolution of defects in bone repair that occur frequently in the elderly. Having the possibility to deliver osteogenic biological factors, such as GH, in situ, thus avoids the unwanted side effects of systemic administration and could have a great therapeutic potential that deserves to be further investigated.

## 8. Conclusions

In summary, besides the well-known endocrine effects of GH, recent research has shown that GH can also play an important role in the commitment of MSCs. During aging, the reduced number and functionality of MSCs are thought to negatively impact the repair/regeneration of damaged tissues. The local delivery of MSCs is associated with trophic factors favoring osteogenesis and might improve the repair process and, consequently, the quality of life of the senile population. However, the lack of standardization of isolation methods and cell culture protocols needs to be overcome in order to eliminate the substantial variability in cell quality. Moreover, the absence of a universal marker remains a major challenge for consistent MSC characterization and their use in clinics. Nevertheless, the combination of polymer scaffolds with MSCs and GH has great potential for future clinical applications. As the number of studies addressing this issue are still few, further studies are needed to address the feasibility of this therapeutic approach in humans in greater detail.

## Abbreviations

AT-MSC	adipose tissue mesenchymal stem cells
bGH	transgenic mice expressing constitutively bovine growth hormone
BM-MSC	bone marrow mesenchymal stem cells
CEBPα	CCAAT enhancer binding protein
ERK	extracellular signal regulated kinase
FABP4	fatty acid-binding protein 4
FAK	focal adhesion kinase
GH	growth hormone
GHBP	growth hormone-binding protein
GHD	growth hormone deficiency
GHR	growth hormone receptor
GHRH	growth hormone releasing hormone
GHRKO	mouse knockout for the growth hormone receptor
IGF I	insulin-like growth factor I
IRS	insulin receptor substrate
JAK2	Janus kinase 2
MSC	mesenchymal stem cell
PLGA	poly(lactic-co-glycolic acid)
PPARγ	peroxisome proliferator-activated receptor γ
PTP	phosphotyrosine phosphatases
rhGH	recombinant human growth hormone
SOCS	suppressors of cytokine signaling
STAT	signal transducers and activators of transcription

## References

[1] Friedenstein A.J., Petrakova K.V., Kurolesova A.I., Frolova G.P. (1968). Heterotopic of bone marrow. Analysis of precursor cells for osteogenic and hematopoietic tissues. Transplantation.

[2] Giustina A., Veldhuis J.D. (1998). Pathophysiology of the neuroregulation of growth hormone secretion in experimental animals and the human. Endocr. Rev..

[3] Anderson L.L., Scanes C.G. (2012). Nanobiology and physiology of growth hormone secretion. Exp. Biol. Med. (Maywood).

[4] Anderson L.L., Jeftinija S., Scanes C.G. (2004). Growth hormone secretion: Molecular and cellular mechanisms and in vivo approaches. Exp. Biol. Med. (Maywood).

[5] Cruz C.R., Smith R.G. (2008). The growth hormone secretagogue receptor. Vitam. Horm..

[6] Wehrenberg W.B., Giustina A. (1992). Basic counterpoint: Mechanisms and pathways of gonadal steroid modulation of growth hormone secretion. Endocr. Rev..

[7] Giustina A., Wehrenberg W.B. (1992). The role of glucocorticoids in the regulation of growth hormone secretion. Trends Endocrinol. Metab..

[8] Harvey S. (2010). Extrapituitary growth hormone. Endocrine.

[9] Pérez-Ibave D.C., Rodríguez-Sánchez I.P., Garza-Rodríguez M.d.L., Barrera-Saldaña H.A. (2014). Extrapituitary growth hormone synthesis in humans. Growth Horm. IGF Res..

[10] Leung D.W., Spencer S.A., Cachianes G., Hammonds R.G., Collins C., Henzel W.J., Barnard R., Waters M.J., Wood W.I. (1987). Growth hormone receptor and serum binding protein: Purification, cloning and expression. Nature.

[11] Clark R.G., Mortensen D.L., Carlsson L.M., Spencer S.A., McKay P., Mulkerrin M., Moore J., Cunningham B.C. (1996). Recombinant human growth hormone (GH)-binding protein enhances the growthpromoting activity of human GH in the rat. Endocrinology.

[12] Amit T., Youdim M.B., Hochberg Z. (2000). Does serum growth hormone (GH) binding protein reflect human GH receptor function?. J. Clin. Endocrinol. Metab..

[13] Baumann G. (2001). Growth hormone binding protein. J. Pediatr. Endocrinol. Metab..

[14] Fisker S., Vahl N., Jorgensen J.O., Christiansen J.S., Orskov H. (1997). Abdominal fat determines growth hormone-binding protein levels in healthy non obese adults. J. Clin. Endocrinol. Metab..

[15] Ballesteros M., Leung K.C., Ross R.J., Iismaa T.P., Ho K.K. (2000). Distribution and abundance of messenger ribonucleic acid for growth hormone receptor isoforms in human tissues. J. Clin. Endocrinol. Metab..

[16] Bergan-Roller H.E., Sheridan M.A. (2018). The growth hormone signaling system: Insights into coordinating the anabolic and catabolic actions of growth hormone. Gen. Comp. Endocrinol..

[17] Landin-Wilhelmens K., Wilhelmens L., Lappas G. (1994). Serum IGF-I in a random population sample of men, and women: Relation to age, sex, smoking habits, coffee consumption and physical activity, blood pressure and concentrations of plasma lipids, fibrinogen, PTH and osteocalcin. Clin. Endocrinol. (Oxf).

[18] Hermann M., Berger P. (2001). Hormonal changes in aging men: A therapeutic indication?. Exp. Gerontol..

[19] Waters M.J. (2016). The growth hormone receptor. Growth Horm. IGF Res..

[20] Brown R.J., Adams J.J., Pelekanos R.A., Wan Y., McKinstry W.J., Palethorpe K., Seeber R.M., Monks T.A., Eidne K.A., Parker M.W. (2005). Model for growth hormone receptor activation based on subunit rotation within a receptor dimer. Nat. Struct. Mol. Biol..

[21] De Vos A.M., Ultsch M., Kossiakoff A.A. (1992). Human growth hormone and extracellular domain of its receptor: Crystal structure of the complex. Science.

[22] Wells J.A. (1996). Binding in the growth hormone receptor complex. Proc. Natl. Acad. Sci. USA.

[23] Birzniece V., Sata A., Ho K.K.Y. (2009). Growth hormone receptor modulators. Rev. Endocr. Metab. Dis..

[24] Ahmed S.F., Farquharson C. (2010). The effect of GH and IGFI on linear growth and skeletal development and their modulation by SOCS proteins. J. Endocrinol..

[25] Bolamperti S., Mrak E., Moro G.L., Sirtori P., Fraschini G., Guidobono F., Rubinacci A., Villa I. (2013). 17β-Estradiol positively modulates growth hormone signaling through the reduction of SOCS2 negative feedback in human osteoblasts. Bone.

[26] Strous G.J., van Kerkhof P., Govers R., Ciechanover A., Schwartz A.L. (1996). The ubiquitin conjugation system is required for ligand-induced endocytosis and degradation of the growth hormone receptor. Embo J..

[27] Strous G.J., van Kerkhof P., Govers R., Rotwein P., Schwartz A.L. (1997). Growth hormone-induced signal transduction depends on an intact ubiquitin system. J. Biol. Chem..

[28] Zhu T., Goh E.L., Graichen R., Ling L., Lobie P.E. (2001). Signal transduction via the growth hormone receptor. Cell. Signal..

[29] Vanderkuur J.A., Butch E.R., Waters S.B., Pessin J.E., Guan K.L., Carter-Su C. (1997). Signaling molecules involved in coupling growth hormone receptor to MAP kinase activity. Endocrinol..

[30] Piwien-Pilipuk G., MacDougald O., Schwartz J. (2002). Dual regulation of phosphorylation and dephosphorylation of C/EBPbeta modulate its transcriptional activation and DNA binding in response to growth hormone. J. Biol. Chem..

[31] Yamauchi T., Kaburagi Y., Ueki K., Tsuji Y., Stark G.R., Kerr I.M., Tsushima T., Akanuma Y., Komuro I., Tobe K. (1998). Growth hormone and prolactin stimulate tyrosine phosphorylation of insulin receptor substrate-1, -2, and -3, their association with p85 phosphatidylinositol 3-kinase (PI3-kinase), and concomitantly PI3-kinase activation via JAK2 kinase. J. Biol. Chem..

[32] Zhu T., Goh E.L., Lobie P.E. (1998). Growth hormone stimulates the tyrosine phosphorylation and association of p125 focal adhesion kinase (FAK) with JAK2. FAK is not required for Stat-mediated transcription. J. Biol. Chem..

[33] Ryu H., Lee J.H., Kim K.S., Jeong S.M., Kim P.H., Chung H.T. (2000). Regulation of neutrophil adhesion by pituitary growth hormone accompanies tyrosine phosphorylation of Jak2, p125FAK, and paxillin. J. Immunol..

[34] Takahashi M.O., Takahashi Y., Iida K., Okimura Y., Kaji H., Abe H., Chihara K. (1999). Growth hormone stimulates tyrosine phosphorylation of focal adhesion kinase (p125(FAK)) and actin stress fiber formation in human osteoblast-like cells, Saos2. Biochem. Biophys. Res. Commun..

[35] Schlaepfer D.D., Hunter T. (1998). Integrin signaling and tyrosine phosphorylation: Just the FAKs?. Trends Cell. Biol..

[36] Herrington J., Smit L.S., Schwartz J., Carter-Su C. (2000). The role of STAT proteins in growth hormone signaling. Oncogene Res..

[37] Waxman D.J., O’Connor C. (2006). Growth hormone regulation of sex dependent liver gene expression. Mol. Endocrinol..

[38] Chia D.J., Ono M., Woelfle J., Schlesinger-Massart M., Jiang H., Rotwein P. (2006). Characterisation of distinct Stat5b binding sites that mediate growth hormone-stimulated IGF-I gene transcription. J. Biol. Chem..

[39] Rowlinson S.W., Yoshizato H., Barclay J.L., Brooks A.J., Behncken S.N., Kerr L.M., Millard K., Palethorpe K., Nielsen K., Clyde-Smith J. (2008). An agonist-induced conformational change in the growth hormone receptor determines the choice of signalling pathway. Nat. Cell Biol..

[40] Lincoln D.T., Sinowatz F., Kölle S., Takahashi H., Parsons P., Waters M.J. (1999). Up-regulation of growth hormone receptor immunoreactivity in human melanoma. Anticancer Res..

[41] García-Caballero T., Mertani H.M., Lambert A., Gallego R., Fraga M., Pintos E., Forteza J., Chevallier M., Lobie P.E., Vonderhaar B.K. (2000). Increased expression of growth hormone and prolactin receptors in hepatocellular carcinoma. Endocrine.

[42] Conway-Campbell B.L., Wooh J.W., Brooks A.J., Gordon D., Brown R.J., Lichanska A.M., Chin H.S., Barton C.L., Boyle G.M., Parsons P.G. (2007). Nuclear targeting of the growth hormone receptor results in dysregulation of cell proliferation and tumorigenesis. Proc. Natl. Acad. Sci. USA.

[43] Giustina A., Mazziotti G., Canalis E. (2008). Growth hormone, insulin-like growth factors, and the skeleton. Endocr. Rev..

[44] Mrak E., Villa I., Lanzi R., Losa M., Guidobono F., Rubinacci A. (2007). Growth hormone stimulates osteoprotegerin expression and secretion in human osteoblast-like cells. J. Endocrinol..

[45] Menagh P.J., Turner R.T., Jump D.B., Wong C.P., Lowry M.B., Yakar S., Rosen C.J., Iwaniec U.T. (2010). Growth hormone regulates the balance between bone formation and bone marrow adiposity. J. Bone Min. Res..

[46] Olney R.C. (2003). Regulation of bone mass by growth hormone. Med. Pediatr. Oncol..

[47] Ohlsson C., Bengtsson B.A., Isaksson O.G., Andreassen T.T., Slootweg M.C. (1998). Growth hormone and bone. Endocr. Rev..

[48] Murray P.G., Clayton P.E., Feingold K.R., Anawalt B., Boyce A., Chrousos G., Dungan K., Grossman A., Hershman J.M., Kaltsas G., Koch C., Kopp P. (2000). Disorders of Growth Hormone in Childhood. Endotext [Internet].

[49] Richmond E., Rogol A.D. (2016). Treatment of growth hormone deficiency in children, adolescents and at the transitional age. Best Pr. Res. Clin. Endocrinol. Metab..

[50] Shi J., Sekhar R.V., Balasubramanyam A., Ellis K., Reeds P.J., Jahoor F., Sharma M.D. (2003). Short- and long-term effects of growth hormone (GH) replacement on protein metabolism in GH-deficient adults. J. Clin. Endocrinol. Metab..

[51] Chikani V., Ho K.K.Y. (2014). Action of GH on skeletal muscle function: Molecular and metabolic mechanisms. J. Mol. Endocrinol..

[52] Bonora M., Patergnani S., Rimessi A., De Marchi E., Suski J.M., Bononi A., Giorgi C., Marchi S., Missiroli S., Poletti F. (2012). ATP synthesis and storage. Purinergic Signal..

[53] Sotiropoulos A., Ohanna M., Kedzia C., Menon R.K., Kopchick J.J., Kelly P.A., Pende M. (2006). Growth hormone promotes skeletal muscle cell fusion independent of insulin-like growth factor 1 up-regulation. Proc. Natl. Acad. Sci. USA.

[54] Wang J., Zhou J., Cheng C.M., Kopchick J.J., Bondy C.A. (2004). Evidence supporting dual, IGF-1 independent and IGF-1 dependent, roles for GH in promoting longitudinal bone growth. J. Endocrinol..

[55] Freda P.U., Shen W., Heymsfield S.B., Reyes-Vidal C.M., Geer E.B., Bruce J.N., Gallagher D. (2008). Lower visceral and subcutaneous but higher intermuscular adipose tissue depots in patients with growth hormone and insulin-like growth factor I excess due to acromegaly. J. Clin. Endocrinol. Metab..

[56] Benencia F., Harshman S., Duran-Ortiz S., Lubbers E.R., List E.O., Householder L., Alnaeeli M., Liang X., Welch L., Kopchick J.J. (2015). Male bovine GH transgenic mice have decreased adiposity with an adipose depot-specific increase in immune cell populations. Endocrinology.

[57] Berryman D.E., List E.O., Kohn D.T., Coschigano K.T., Seeley R.J., Kopchick J.J. (2006). Effect of growth hormone on susceptibility to diet-induced obesity. Endocrinology.

[58] Chaves V.E., Mesquita Junior F., Bertolini G.L. (2013). The metabolic effects of growth hormone in adipose tissue. Endocrine.

[59] Bredella M.A., Karastergiou K., Bos S.A., Gerweck A.V., Torriani M., Fried S.K., Miller K.K. (2017). GH administration decreases subcutaneous abdominal adipocyte size in men with abdominal obesity. Growth Horm IGF Res..

[60] Rasmussen M.H. (2010). Obesity, growth hormone and weight loss. Mol. Cell. Endocrinol..

[61] Lam K.S., Xu A., Tan K.C., Wong L.C., Tiu S.C., Tam S. (2004). Serum adiponectin is reduced in acromegaly and normalized after correction of growth hormone excess. J. Clin. Endocrinol. Metab..

[62] Silha J.V., Krsek M., Hana V., Marek J., Jezkova J., Weiss V., Murphy L.J. (2003). Perturbations in adiponectin, leptin and resistin levels in acromegaly: Lack of correlation with insulin resistance. Clin. Endocrinol..

[63] Møller N., Jørgensen J.O. (2009). Effects of growth hormone on glucose, lipid and protein metabolism in human subjects. Endocr. Rev..

[64] Roemmler J., Gockel A., Otto B., Bidlingmaier M., Schopohl J. (2012). Effects on metabolic variables after 12-month treatment with a new once a week sustained-release recombinant growth hormone (GH:LB03002) in patients with GH deficiency. Clin. Endocrinol..

[65] Caplan A.I. (1991). Mesenchymal stem cells. J. Orthop. Res..

[66] Caplan A.I. (2005). Review: Mesenchymal stem cells: Cell-based reconstructive therapy in orthopedics. Tissue Eng..

[67] Da Silva Meirelles L., Chagastelles P.C., Nardi N.B. (2006). Mesenchymal stem cells reside in virtually all post-natal organs and tissues. J. Cell. Sci..

[68] Frenette P.S., Pinho S., Lucas D., Scheiermann C. (2013). Mesenchymal stem cell: Keystone of the hematopoietic stem cell niche and a stepping-stone for regenerative medicine. Annu. Rev. Immunol..

[69] Krampera M., Pasini A., Pizzolo G., Cosmi L., Romagnani S., Annunziato F. (2006). Regenerative and immunomodulatory potential of mesenchymal stem cells. Curr. Op. Pharm..

[70] Siegel G., Schäfer R., Dazzi F. (2009). The immunosuppressive properties of mesenchymal stem cells. Transplantation.

[71] Dominici M., Le Blanc K., Mueller I., Slaper-Cortenbach I., Marini F., Krause D., Deans R., Keating A., Prockop D.j., Horwitz E. (2006). Minimal criteria for defining multipotent mesenchymal stromal cells. The International Society for Cellular Therapy position statement. Cytotherapy.

[72] Calloni R., Cordero E.A.A., Henriques J.A.P., Bonatto D. (2013). Reviewing and updating the major molecular markers for stem cells. Stem Cells Dev..

[73] Lv F.-J., Tuan R.S., Cheung K.M.C., Leung V.Y.L. (2014). Concise review: The surface markers and identity of human mesenchymal stem cells. Stem Cells.

[74] Pittenger M.F., Mackay A.M., Beck S.C., Jaiswal R.K., Douglas R., Mosca J.D., Moorman M.A., Simonetti D.W., Craig S., Marshak D.R. (1999). Multilineage potential of adult human mesenchymal stem cells. Science.

[75] Da Silva Meirelles L., Fontes A.M., Caplan A.I. (2009). Mechanisms involved in the herapeutic properties of mesenchymal stem cells. Cytokine Growth Factor Rev..

[76] Kassis I., Zangi L., Rivkin R., Levdansky L., Samuel S., Marx G., Gorodetsky R. (2006). Isolation of mesenchymal stem cells from G-CSF-mobilized human peripheral blood using fibrin microbeads. Bone Marrow Transpl..

[77] Zou Z., Zhang Y., Hao L., Wang F., Liu D., Su Y., Sun H. (2010). More insight into mesenchymal stem cells and their effects inside the body. Exp. Opin. Biol. Ther..

[78] Lin G., Garcia M., Ning H., Banie L., Guo Y.L., Lue T.F., Lin C.S. (2008). Defining stem and progenitor cells within adipose tissue. Stem Cells Dev..

[79] Corselli M., Chen C.W., Crisan M., Lazzari L., Péault B. (2010). Perivascular ancestors of adultmultipotent stem cells. Arter. Thromb. Vasc. Biol..

[80] Fong C.Y., Chak L.L., Biswas A., Tan J.H., Gauthaman K., Chan W.K., Bongso A. (2011). Human Wharton’s jelly stem cells have unique transcriptome profiles compared to human embryonic stem cells and other mesenchymal stem cells. Stem Cell Rev..

[81] Caplan A.I., Dennis J.E. (2006). Mesenchymal stem cells as trophic mediators. J. Cell. Biochem..

[82] Lin P., Correa D., Kean T.J., Awadallah A., Dennis J.E., Caplan A.I. (2014). Serial transplantation and long-term engraftment of intra-arterially delivered clonally derived mesenchymal stem cells to injured bone marrow. Mol. Ther..

[83] Murphy M.B., Moncivais K., Caplan A.I. (2013). Mesenchymal stem cells: Environmentally responsive therapeutics for regenerative medicine. Exp. Mol. Med..

[84] Le Blanc K., Mougiakakos D. (2012). Multipotent mesenchymal stromal cells and the innate immune system. Nat. Rev. Immunol..

[85] Caplan A.I. (2015). Adult mesenchymal stem cells: When, where, and how. Stem Cells Int..

[86] Wagner W., Horn P., Castoldi M., Diehlmann A., Bork S., Saffrich R., Benes V., Blake J., Pfister S., Eckstein V. (2008). Replicative Senescence of Mesenchymal Stem Cells: A Continuous and Organized Process. PLoS ONE.

[87] Sethe S., Scutt A., Stolzing A. (2006). Aging of mesenchymal stem cells. Ageing Res. Rev..

[88] Zhou S., Greenberger J.S., Epperly M.W., Goff J.P., Adler C., Leboff M.S., Glowacki J. (2008). Age-related intrinsic changes in human bone-marrow-derived mesenchymal stem cells and their differentiation to osteoblasts. Aging Cell..

[89] Olarescu N.C., Berryman D.E., Householder L.A., Lubbers E.R., List E.O., Benencia F., Kopchick J.J., Bollerslev J. (2015). GH action influences adipogenesis of mouse adipose tissue-derived mesenchymal stem cells. J. Endocrinol..

[90] Ross S.E., Hemati N., Longo K.A., Bennett C.N., Lucas P.C., Erickson R.L., MacDougald O.A. (2000). Inhibition of adipogenesis by Wnt signalling. Science.

[91] Prestwich T.C., McDougald O.A. (2007). Wnt/β catenin signaling in adipogenesis and metabolism. Curr. Opin. Cell Biol..

[92] Bolamperti S., Signo M., Spinello A., Moro G., Fraschini G., Guidobono F., Rubinacci A., Villa I. (2018). GH prevents adipogenic differentiation of mesenchymal stromal stem cells derived from human trabecular bone via canonical Wnt signaling. Bone.

[93] Jia D., Zheng W., Jiang H. (2018). Growth hormone facilitates 5′-azacytidine-induced myogenic but inhibits 5′-azacytidine-induced adipogenic commitment in C3H10T1/2 mesenchymal stem cells. Growth Horm. IGF Res..

[94] Taylor S.M., Jones P.A. (1979). Multiple new phenotypes induced in 10T1/2 and 3T3 cells treated with 5-azacytidine. Cell.

[95] Huang E., Zhu G., Jiang W., Yang K., Gao Y., Luo Q., Gao J.L., Kim S.H., Liu X., Li M. (2012). Growth hormone synergizes with BMP9 in osteogenic differentiation by activating the JAK/STAT/IGF1 pathway in murine multilineage cells. J. Bone Min. Res..

[96] Dalby M.J., Gadegaard N., Tare R., Andar A., Riehle M.O., Herzyk P., Wilkinson C.D., Oreffo R.O. (2007). The control of human mesenchymal cell differentiation using nanoscale symmetry and disorder. Nat. Mater..

[97] Guicheux J., Gauthier O., Aguado E., Pilet P., Couillaud S., Jegou D., Daculsi G., Heymann D. (1998). Human growth hormone locally released in bone sites by calcium-phosphate biomaterial stimulates ceramic bone substitution without systemic effects: A rabbit study. J. Bone Min. Res..

[98] Tresguerres I.F., Blanco L., Clemente C., Tresguerres J.A. (2003). Effects of local administration of growth hormone in peri-implant bone: An experimental study with implants in rabbit tibiae. Int. J. Oral Maxillofac. Implant..

[99] Wang J.R., Ahmed S.F., Gadegaard N., Meek R.M., Dalby M.J., Yarwood S.J. (2014). Nanotopology potentiates growth hormone signalling and osteogenesis of mesenchymal stem cells. Growth Horm. IGF Res..

[100] Da Silveira Gerzson A., Machado D.C., Marinovic D.R., Pagnoncelli R.M. (2017). Assessment of Adhesion and Proliferation of Bone Marrow Mesenchymal Stem Cells in Polymer Matrices with rhGH. Int. J. Oral Maxillofac. Implants..

